# Unraveling cardiac amyloidosis in Egypt: diagnostic challenges and disease outcomes

**DOI:** 10.1186/s43044-026-00784-x

**Published:** 2026-09-29

**Authors:** Rasha Mohamed Abayazeed, Ingy Etman, Mahmoud Hassanein, Hagar Mahmoud Kamal, Mohamed Elsakhawy, Ahmed Abdelaaty, Maram Allam, Nouran Soror, Ashraf Elghandour, Mohamed H. Elbahoty, Sara Salama, Osama Mohammed Refai, Noha Elkholy

**Affiliations:** 1 Cardiology Department, Faculty of Medicine, Alexandria University, Alexandria, Egypt; 2 Pathology Department, Faculty of Medicine, Alexandria University, Alexandria, Egypt; 3 Hematology Unit, Department of Internal Medicine, Alexandria University, Alexandria, Egypt; 4 Department of Pathology, University of Alabama at Birmingham Hospital, Birmingham, USA; 5 Department of Neurology, Alexandria University, Alexandria, Egypt; 6 Nephrology Unit, Department of Internal Medicine, Alexandria University, Alexandria, Egypt

**Keywords:** AA amyloidosis, Egypt, Immunoglobulin light-chain amyloidosis, Echocardiography, Patient care

## Abstract

**Background:**

Cardiac amyloidosis is an infiltrative cardiomyopathy caused by extracellular deposition of insoluble amyloid fibrils. Despite the growing global recognition of cardiac amyloidosis and the recent development of targeted therapeutic strategies for specific amyloidosis subtypes, demographic and epidemiological data regarding cardiac amyloidosis in Egypt remain limited. The aim of the study was to describe the demographic and clinical characteristics of cardiac amyloidosis in Egyptian patients; and to identify the diagnostic challenges associated with cardiac amyloidosis and the disease-related outcomes.

**Results:**

Among 84 patients initially evaluated for suspected cardiac amyloidosis at tertiary care facility in Egypt, 70 were confirmed to have the disease. The mean interval from symptom onset to diagnosis was 11.86 months. AL amyloidosis was the most common subtype, followed by AA amyloidosis. ATTR amyloidosis accounted for only 4.3% of cases, while the specific amyloidosis subtype could not be identified in 21.4% of patients. New complications developed in 21.4% of patients, with renal complications being the most common. The study observed a high mortality rate, with 48.6% of patients dying during the study period.

**Conclusions:**

This tertiary care center study of cardiac amyloidosis in Egyptian patients demonstrated a substantial diagnostic delay and poor clinical outcomes. In our study cohort, the most common subtype was AL amyloidosis and nearly half of the study population died during the study period, highlighting the advanced stage of disease at presentation and its grave prognosis. These findings underscore the need for greater clinical awareness, streamlined diagnostic pathways and improved access to comprehensive diagnostic modalities to ensure timely diagnosis and optimal patient care.

## Introduction

Amyloidosis is a clinical disorder caused by pathological deposition of insoluble fibrils, with beta-pleated sheet configuration, in the interstitium of various organs leading to progressive organ dysfunction [1, 2]. Amyloidosis affects both sexes and individuals of any age. As a multisystem disorder with diverse, often nonspecific clinical manifestations of variable severity, its diagnosis is frequently delayed, and a substantial number of cases are likely to remain undiagnosed [3–6]. Despite the growing global recognition of cardiac amyloidosis and the recent development of targeted therapeutic strategies for specific amyloidosis subtypes [7–10], demographic and epidemiological data regarding cardiac amyloidosis in Egypt remain limited and it is still considered a rare and incurable disease by many physicians [11, 12]. 

We considered initiating an observational study to describe the demographic and clinical characteristics of cardiac amyloidosis in Egyptian patients and to identify the diagnostic challenges associated with cardiac amyloidosis and the disease-related outcomes.

## Methods

### Study design

This was a single-center, prospective, observational study that included all patients with suspected cardiac amyloidosis, according to the current recommendations [4, 8, 13], presented to or referred to Alexandria Main University Hospital from November 2021 to February 2026. No specific exclusion criteria except for age (patients had to be older than 18 years). Final diagnosis was based on the results of all investigations alongside the results of histological examination of available biopsy specimens. Standard management of patients was the diagnostic and therapeutic interventions performed according to the current guidelines. No specific protocols or recommendations for evaluation, management, and/or treatment were put forth during this observational study.

### Clinical assessment and laboratory investigations

Demographic data and clinical characteristics were collected for all enrolled patients with special emphasis on red flags suggestive of systemic amyloidosis [8, 13]. Standard 12 lead-electrocardiogram (ECG) was performed for assessment of QRS voltage, detection of repolarization abnormalities, arrythmias or conduction disease. Laboratory investigations included complete blood count, renal function tests, troponin I or T and pro-B-type brain natriuretic peptides (proBNP) and assessment for proteinuria. Serum and urine protein electrophoresis with immunofixation and ratio of serum free kappa: lambda light chains were done in all patients. Further laboratory investigations were performed based on the patient’s clinical profile.

### Echocardiography

All studies were performed on EPIQ CVx machine (Philips Medical Systems). Comprehensive transthoracic echocardiography was performed in all participants and included evaluation of left ventricular (LV) wall thickness, LV systolic and diastolic function, left atrial (LA) volume, right ventricular (RV) wall thickness and systolic function, and the presence and severity of valvular heart disease. Speckle tracking echocardiography (STE) was performed for assessment of LV global longitudinal strain and detection of apical sparing; assessment LA strain and RV free wall strain were also done when feasible.

### Other investigations

Further investigations were performed according to the availability and suspected amyloidosis subtype and included:

#### Contrast enhanced cardiac magnetic resonance imaging (CE-CMR)

features typical of cardiac amyloidosis were defined as LV wall thickness > upper limit normal (ULN) for sex, global extracellular volume (ECV) > 0.40, diffuse late gadolinium enhancement (LGE) and abnormal myocardium and blood pool gadolinium kinetics [14]. 

#### Technetium-99 m pyrophosphate (PYP) scintigraphy with concomitant SPECT images

positive study was defined as grade 2 or 3 myocardial uptake of radiotracer [14]. It was used for non-invasive diagnosis of transthyretin amyloidosis (ATTR) based on positive study+ negative serum free light chains and negative serum and urine immunofixation+ echocardiographic/CMR criteria [8, 10, 13, 14]. 

#### Tissue biopsy (surrogate or affected organ)

Histopathology: formalin- fixed paraffin embedded biopsies were stained by H&E and Congo red stains and examined under polarized light microscope [15, 16]. Immunohistochemistry: immunohistochemical staining using anti Kappa, Lambda and AA (amyloid A) protein antibodies was performed using ROCHE Benchmark Ultra Immunohistochemical auto stainer.

### Follow up data

Follow-up was performed as clinically indicated. New complications or mortality among the study population were recorded.

### Statistical analysis

Data were fed into the computer and analyzed using IBM SPSS software package version 27.0. (Armonk, NY: IBM Corp, released 2020). Categorical data were summarized as numbers and percentages. For continuous data, normality was assessed using the **Shapiro-Wilk test**, Quantitative data were described using range (minimum and maximum), mean ± standard deviation (SD.) and median with interquartile range (IQR). Significance of the obtained results was judged at the 5% level, all tests were two-tailed.

#### The used tests were

1. **Chi-square test**: For categorical variables, to compare between two groups
2. **Fisher’s Exact or Monte Carlo correction: **Correction for chi-square when more than 20% of the cells have expected count <5 , Fisher Exact correction test was applied was applied for 2×2 tables while Monte Carlo correction test was used for tables larger than 2×2
3. **Student t-test:** For normally distributed quantitative variables, to compare between two studied groups
4. **Mann Whitney test: **For non-normally distributed quantitative variables, to compare between two studied groups

## Results

A total of 84 patients with suspected cardiac amyloidosis were initially enrolled in the study. Fourteen patients were subsequently excluded: 11 because further investigations established an alternative diagnosis, 2 who declined further assessment, and 1 who died before the diagnosis could be confirmed. Consequently, the final study cohort comprised 70 patients with confirmed cardiac amyloidosis.

### Demographic data and clinical characteristics of study population

The mean age of the study population was 54.37 ± 13.85 years (range, 19–85 years), with 77% of patients being younger than 65 years. Males constituted 64.3% of the study population, while 34.3% had a history of smoking. Renal impairment was the most prevalent comorbidity, affecting 62.9% of patients, followed by a history of hypotension in 47.1%. Neurological manifestations were reported in 27.1% of cases and included autonomic dysfunction, peripheral neuropathy and surgically treated or current bilateral carpal tunnel syndrome (CTS), with these manifestations frequently overlapping in the same patient. The demographic characteristics and baseline medical history of the study population are presented in Table 1. At presentation, 77% of patients were receiving pharmacological therapy for either heart failure or associated comorbid conditions. Details of the patients’ medication history are also summarized in Table 1.

**Table 1:** Demographic data, comorbidities and drug history of the study population

Demographic data and comorbidities	Number (%)
**Age (years)**	
Mean± SD	54.37 ± 13.85
**Gender**	
Males	45 (64.3)
Females	25 (35.7)
**Smoking **	
None	46 (65.7)
Current smoker	14 (20.0)
Ex-smoker	10 (14.3)
**Diabetes Mellitus**	13 (18.6)
**Hypertension**	9 (12.9)
**Hypotension**	33 (47.1)
**Normotensive despite previously hypertensive**	8 (11.4)
**Coronary artery disease**	3 (4.3)
**Previous TIA or CVS**	4 (5.7)
**Venous thromboembolism (DVT/PE)**	6 (8.6)
**Renal impairment**	44 (62.9)
**Maintenance hemodialysis**	9 (12.9)
**Bilateral carpel tunnel syndrome (current or surgically treated)**	3 (4.3)
**Peripheral polyneuropathy**	9 (12.8)
**Autonomic dysfunction**	12 (17.1)
**Others**	25 (40.9)
Multiple myeloma	3 (4.3)
Plasma cell leukemia	1 (1.4)
Peripheral arterial disease	1 (1.4)
Liver disease (bilharziasis, liver cirrhosis)	6 (8.6)
Familial Mediterranean fever	4 (5.7)
Pulmonary disease (COPD, ILF)	2 (2.9)
Pregnancy	1 (1.4)
Paroxysmal AF	2 (2.9)
Thyroid disease	9 (12.9)
Collagenic disease	1 (1.4)
Complete heart block	1 (1.4)
Chronic anemia	3 (4.3)
Infective endocarditis	1 (1.4)
**Drug history**	
None	16 (22.9)
Diuretics (loop diuretics± MRA)	39 (55.7)
Beta blockers	16 (22.9)
Calcium channel blockers	3 (4.3)
Ivabradine	4 (5.7)
Antiplatelets	11 (15.7)
Anticoagulants (VKAs, DOACs)	6 (8.6)
Immunosuppressants	7 (10.0)
Oral hypoglycemic drugs	8 (11.4)
Statins	10 (14.3)
Midodrine	4 (5.7)
ACEIs/ARBs/ARNI	7 (10.0)
Chemotherapy	3 (4.3)
Colchicine	6 (8.6)
SGLT2 inhibitors	6 (8.6)
Drugs for thyroid dysfunction	6 (8.6)
Amiodarone	1 (1.4)
Digoxin	2 (2.9)

ACEIs: angiotensin-converting enzyme inhibitors; AF: atrial fibrillation; ARBs: angiotensin receptor blockers; ARNIs: angiotensin receptor-neprilysin inhibitors; COPD: chronic obstructive pulmonary disease; CVS: cerebrovascular stroke; DOACs: direct oral anticoagulants; DVT: deep venous thrombosis; ILF: interstitial lung fibrosis; MRA: mineralocorticoid receptor antagonist; PE: pulmonary embolism; SD: standard deviation; SGLT2: sodium-glucose cotransporter 2 ;TIA: transient ischemic attack; VKA: vitamin K antagonist

Regarding clinical symptoms, dyspnea, with or without orthopnea, was the most common presenting symptom in 92.9% of cases, with most patients being in New York Heart Association (NYHA) III-IV functional class (61.5%), followed by lower limb (LL) oedema in 67.1% of patients. Other symptoms included palpitations, chest pain, dizziness, syncope, generalized fatigue, weight loss and gastrointestinal tract manifestations as detailed in Table 2.

**Table 2:** Symptoms and clinical examination of the study population

Symptoms	No (%)
**Dyspnea** NYHA class I NYHA class II NYHA class III-IV	65 (92.9) 1 (1.5) 24 (36.9) 40 (61.5)
**Orthopnea**	24 (34.3)
**Lower limb oedema**	47 (67.1)
**Abdominal distension (ascites)**	13 (18.6)
**Palpitations**	15 (21.4)
**Dizziness**	20 (28.6)
**Syncope**	14 (20.0)
**Others** Weight loss Body aches Generalized fatigue Chest pain GIT manifestations (diarrhea, anorexia, vomiting, abdominal pain, hematemesis and melena) Hoarseness of voice Recurrent pleural effusion	41 (58.6) 10 (14.3) 3 (4.3) 11 (15.7) 9 (12.9) 11 (15.7) 2 (2.9) 1 (1.4)
**Clinical examination**: BMI (kg/m2): mean ± SD Systolic blood pressure (mmHg): mean ± SD Diastolic blood pressure (mmHg): mean ± SD Heart rate (bpm): mean ± SD Orthopnea Periorbital purpura Macroglossia Congested neck veins Carpal tunnel syndrome Polyneuropathy Autonomic dysfunction Skin bruising or discoloration Lower limb oedema Ascites Hepatomegaly Splenomegaly Pleural effusion	26 0.07 ± 5.38 107.86 ± 17.35 67.43 ± 10.17 83.27 ± 13.82 11 (15.7) 3 (4.3) 4 (5.7) 41 (58.6) 2 (2.9) 11 (15.7) 17 (24.3) 6 (8.6) 47 (67.1) 12 (17.1) 15 (21.4) 5 (7.1) 36 (51.4)

BMI: body mass index; GIT: gastro-intestinal tract; NYHA: New York heart association

Upon clinical examination, mean systolic and diastolic BP were 107.86 ± 17.35 and 67.43 ± 10.17 mmHg respectively; and mean HR was 83.27 ± 13.82 bpm. Signs of systemic congestion were the most common findings including congested neck veins, bilateral LL oedema and ascites in 58.6%, 67.1% and 17.1% of patients respectively. Orthopnea was found in 15.7% of patients and evidence of neurological disease in the form of autonomic dysfunction, peripheral polyneuropathy and bilateral carpal tunnel syndrome was found in 24.3%, 15.7% and 2.9% of patients respectively. Signs specific to AL type, namely periorbital purpura, macroglossia and skin bruising were found in 4.3%, 5.7% and 8.6% of cases respectively. Clinical examination findings are presented in Table 2.

Regarding resting ECG, 91.4% of patients were in sinus rhythm. The most prevalent ECG finding was low/decreased QRS voltage to degree of LV thickness in 75.7% of cases followed by pseudo-infarction pattern in 51.4% of patients. Detailed ECG findings are summarized in Table 3.

**Table 3:** Baseline electrocardiographic and laboratory findings in the study population

ECG Findings	No (%)
**Rhythm** Sinus rhythm Atrial fibrillation Paced rhythm	64 (91.4) 5 (7.1) 1 (1.4)
**QRS duration** (msec) mean ± SD **QTc interval** (msec) mean ± SD	84.86 ± 20.69 449.6 ± 40.91
**Long QTc**	19 (27.1)
**Conduction abnormalities** First degree AV block LBBB IVCD Complete heart block	11 (17.1) 2 (2.9) 2 (2.9) 1 (1.4)
**Low/ decreased QRS voltage to degree of LV thickness**	53 (75.7)
**Pseudo-infarction pattern**	36 (51.4)
**Other ECG findings** PACs PVCs Inverted T wave in inferolateral leads Inverted T wave in lateral leads ST segment depression in lateral leads LVH voltage criteria	2 (2.9) 5 (7.1) 6 (8.6) 7 (10.0) 1 (1.4) 3 (4.3)
**Laboratory investigations**	
Hemoglobin (g/dl) : mean ± SD	11.11 ± 2.19
WBCs (x10*3c cells/mm3) :mean ± SD	7.40 ± 2.77
Platelets (x10*3c cells/mm3): mean ± SD	245.6 ± 100.4
Renal impairment (reduced eGFR &/or proteinuria)	56 (80.0)
Urea (mg/dl): mean ± SD	76.22 ± 45.57
Creatinine (mg/dl): mean ± SD	3.42 ± 4.8
eGFR (ml/min): mean ± SD	51.61 ± 30.64
Proteinuria present	47 (67.1)
Elevated cardiac troponin	18 (25.7)
Pro-BNP (pg/ml): mean ± SD	14146.5 ± 11228.6
FLC ratio: median (IQR) Abnormal FLC ratio	0.69 (0.13–1.13) 35 (50.0)
Paraproteins in serum &/or urine protein immunofixation	35 (50.0)
Serum amyloid A (mg/l) (56 patients): median (IQR)	28.80 (7.75–78.35)

AV: atrio-ventricular; ECG: electrocardiogram; eGFR: estimated glomerular filtration rate; FLC: free light chain; IVCD: intraventricular conduction delay; IQR: interquartile range; LV: left ventricle; LVH: left ventricular hypertrophy; LBBB: left bundle branch block; PACs: premature atrial contractions; Pro-BNP: pro-brain natriuretic peptide; PVCs: premature ventricular contractions; SD: standard deviation; QTc: heart-rate corrected QT interval; WBCs: white blood cells

### Laboratory investigations

Renal impairment, defined by a reduced estimated glomerular filtration rate (eGFR) and/or the presence of proteinuria, was observed in 80% of the study population. Cardiac troponin was positive in 25.7% of patients, while the mean pro-BNP level was 14146.5 ± 11228.6 pg/ml. An abnormal serum free kappa: lambda light chain ratio was detected in 50% of patients and serum &/or urine protein immunofixation identified paraproteins in 50% of cases. Serum amyloid A levels were available for 56 patients with median value of 28.8 mg/L ranging from 2.9 to 238 mg/L. Laboratory investigations findings are presented in Table 3.

### Echocardiography findings

**Fig. 1:**
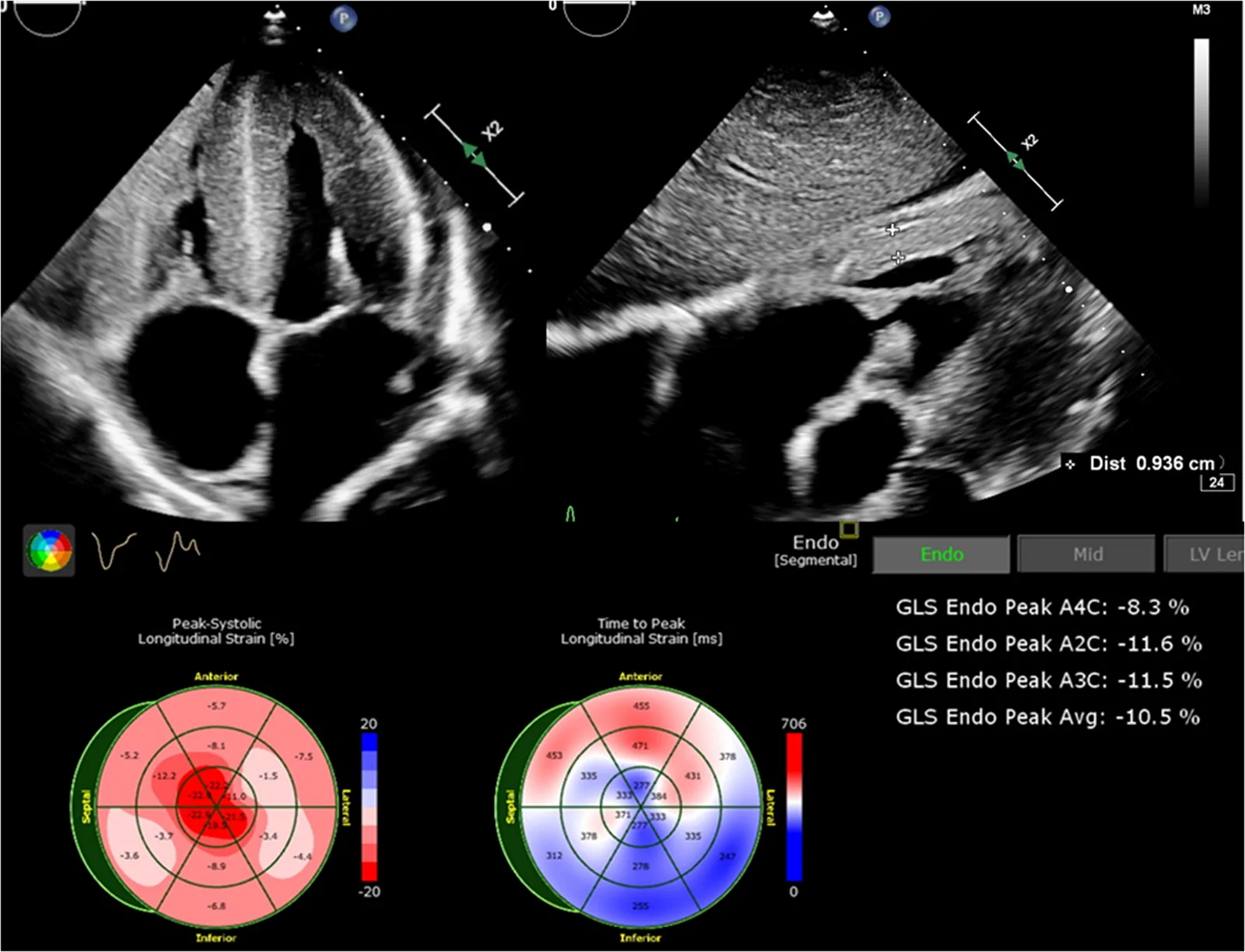
Echocardiographic study in a patient with AL amyloidosis. **A**) Apical 4 chamber view showing severe left ventricular hypertrophy, thickened valves and interatrial septum and a rim of pericardial effusion **B**) Subcostal view showing moderate right ventricular hypertrophy and **C**) Strain analysis of LV showing reduced global longitudinal strain (-10.5%) with relative apical sparing (cherry on top) pattern. AL: amyloid light chain; LV: left ventricle

**Fig. 2:**
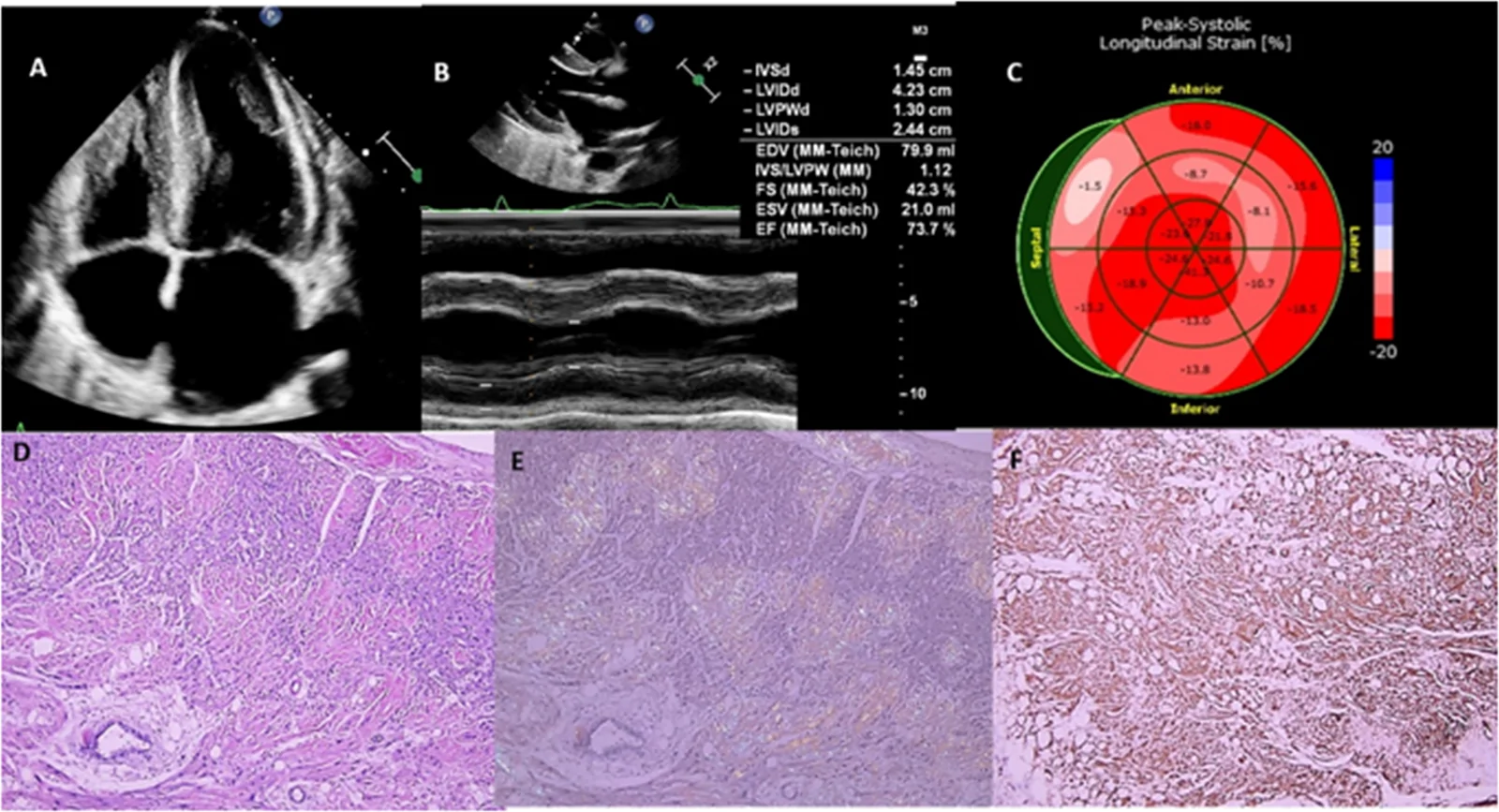
Diagnosis in a patient with AA type (**A**) & (**B**) 2D and M-mode echocardiography showing mild LVH, thickened interatrial septum and minimal pericardial effusion, (**C**) STE showing preserved GLS (-18%) but with relative apical sparing of global LS ratio (average of apical LS/average of combined mid+basal LS > 1). (**D**) & (**E**) Congo red stained section from the received cardiac specimen revealed Salmon pink coloration extracellularly in between the cardiomyocytes and in blood vessels walls, that gave apple green birefringence when examined by a polarized light microscope confirming Amyloid nature of this substance, (**F**) immunohistochemically stained section for anti- Amyloid associated protein antibody (Anti-AA antibody) revealing positivity extracellularly in between the cardiomyocytes and in blood vessels wall. AA: Serum amyloid A amyloidosis; GLS: global longitudinal strain; LS: longitudinal strain; LVH: left ventricular hypertrophy

Pericardial effusion was observed in 88.6% of the study population and was predominantly minimal in severity. Resting left ventricular diastolic dysfunction was identified in 92.9% of patients, with a mean E/e′ ratio of 19.47 ± 8.47. The mean interventricular septal thickness at end-diastole (IVSd) was 15.01 ± 2.44 mm, while the mean left ventricular posterior wall thickness (PWT) was 12.91 ± 2.47 mm, yielding a mean relative wall thickness of 0.61 ± 0.15. The mean left ventricular ejection fraction (LVEF) was 50.79 ± 8.31%. The mean left atrial volume index (LAVi) was 45.12 ± 14.55 mL/m². Right ventricular hypertrophy (RVH) was present in 80% of patients, with a mean right ventricular end-diastolic free wall thickness of 6.06 ± 1.60 mm. Assessment of right ventricular systolic function demonstrated a mean tricuspid annular plane systolic excursion (TAPSE) of 1.65 ± 0.36 cm and a mean tissue Doppler-derived tricuspid annular systolic velocity (RV S′) of 9.95 ± 2.37 cm/s. By speckle tracking echocardiography (STE), mean LV GLS was − 10.49 ± 4.17% with relative apical sparing pattern found in 82.85% of cases (Figs. 1 and 2). Mean LA reservoir strain was 12.99 ± 8.91% while mean RV free wall strain was − 14.87 ± 5.84%. Detailed echocardiographic findings are summarized in Table 4.

**Table 4:** Echocardiographic findings in the study population

Echocardiographic findings	Number (%) or mean ± SD
**Pericardial effusion** No *n* (%) Minimal *n* (%) Mild *n* (%) Moderate *n* (%) Large *n* (%)	8 (11.4) 34 (48.6) 23 (32.9) 4 (5.7) 1 (1.4)
LVEF (%) Mean ± SD	50.79 ± 8.31
IVSd (mm) Mean ± SD	15.01 ± 2.44
LV PW thickness (mm) Mean ± SD	12.91 ± 2.47
Relative wall thickness Mean ± SD	0.61 ± 0.15
LV mass index (g/m2) Mean ± SD	145.78 ± 50.52
LVIDd (mm) Mean ± SD	46.16 ± 7.24
LVIDs (mm) Mean ± SD	33.32 ± 6.94
LVEDVi (ml/m2) Mean ± SD	48.51 ± 14.54
LVESVi (ml/m2) Mean ± SD	24.25 ± 8.87
Left atrial diameter (mm) Mean ± SD	45.31 ± 5.82
LAVi (ml/m2) Mean ± SD	45.12 ± 14.55
Peak E wave velocity (cm/sec) Mean ± SD	89.39 ± 22.12
Peak A wave velocity (cm/sec) (*n* = 62) Mean ± SD	49.76 ± 20.17
Septal e’(cm/sec) Mean ± SD	4.54 ± 1.76
Lateral e’(cm/sec) Mean ± SD	6.05 ± 2.35
E/e’ ratio Mean ± SD	19.47 ± 8.47
Deceleration time (msec) Mean ± SD	155.79 ± 41.24
Resting diastolic dysfunction grade No n (%) Grade I n (%) Grade II n (%) Grade III n (%) Couldn’t specify grade (AF or fused E/A waves) n (%)	5 (7.1) 6 (8.6) 28 (40.0) 23 (32.9) 8 (11.4)
RVH present n (%)	56 (80.0)
RV wall thickness (mm) Mean ± SD	6.06 ± 1.60
TAPSE (cm) Mean ± SD	1.65 ± 0.36
TDI RV S wave (cm/m2) Mean ± SD	9.95 ± 2.37
**Left sided valvular disease** No n (%) Mild mitral regurgitation n (%) Moderate mitral regurgitation n (%) Severe mitral regurgitation n (%) Mild aortic regurgitation n (%) Moderate aortic stenosis n (%)	34 (48.6) 21 (30) 13 (18.6) 1 (1.4) 2 (2.9) 1 (1.4)
Tricuspid valve regurgitation No n (%) Trace n (%) Mild n (%) Moderate n (%) Severe n (%)	20 (28.6) 17 (24.3) 20 (28.6) 9 (12.9) 4 (5.7)
Estimated resting PASP (mmHg) Mean ± SD	42.98 ± 10.93
**Speckle tracking echocardiography** Relative apical sparing present n (%) LV GLS (%) mean ± SD LA reservoir strain (LASr) (%) mean ± SD (*n* = 67) LA conduit (LAScd) (%) mean ± SD (*n* = 67) LA contraction (LASct) (%) mean ± SD (*n* = 67) RV free wall strain (%) mean ± SD (*n* = 65)	58 (82.8) -10.49 ± 4.17 12.99 ± 8.91 -8.48 ± 6.13 -5.25 ± 5.02 -14.87 ± 5.84

AF: atrial fibrillation; GLS: global longitudinal strain; IVSD: interventricular septal thickness in diastole; ; LA: left atrium; LAVI: left atrial volume indexed; LVEF: left ventricular ejection fraction ; LVEDVi: left ventricular end diastolic volume indexed; LVESVi: left ventricular end systolic volume indexed; LVIDd: left ventricular internal diameter in diastole; LVIDs: left ventricular internal diameter in systole; PASP: pulmonary artery systolic pressure; PW: posterior wall RV: right ventricle; RVH: right ventricular hypertrophy; TAPSE: tricuspid annular plane systolic excursion; TDI: tissue doppler imaging

### Other investigations

1. **Contrast enhanced cardiac magnetic resonance imaging (CE-CMR)**: It was performed in 14 patients (20%) with findings typical of cardiac amyloidosis in all of them.
2. **Technetium PYP scintigraphy with SPECT**: It was done in 10 patients (14.3%). It yielded positive results in 4 patients (40%), negative results in 5 cases (50%) and was inconclusive in one patient (10%).

#### Tissue biopsy

- **Bone marrow biopsy/aspirate with flow cytometry:** It was done in 44 patients and revealed normal findings in 4 cases (9.1%), clonal plasma cells <10% in 13 cases (29.5%) and multiple myeloma (MM) in 27 cases (61.4%). Congo-red staining was performed on 21 samples and was positive in 17 cases (38.6%).
- **Renal biopsy with immunohistochemistry:** It was performed in 19 cases (27.1%) of cases and was positive for amyloidosis in all of them.
- **Abdominal fat biopsy: **It was done in 22 patients (31.4%) and was positive for amyloidosis in 13 cases (59.1%).3.
- **Rectal biopsy: **It was performed in one patient (1.4%) and was positive for amyloidosis.
- **Cardiac biopsy: **Intra-operative cardiac biopsy was done in one patient (1.4%) and was positive for cardiac amyloidosis (Fig. 2).
- **Other tissue biopsies:** Other tissue biopsies, including gastric, ileal, colonic, uterine, and thyroid biopsies, were performed in 7.1% of cases, and all were positive for amyloid deposition.

### Specific amyloidosis subtype and treatment

A specific amyloidosis subtype was identified in 78.6% of patients. The most common subtype was AL amyloidosis, accounting for 55.7% (39/70) of cases. Among patients with AL amyloidosis, 35.9% (14/39) had isolated AL disease, while 64.1% (25/39) had AL amyloidosis associated with multiple myeloma (MM). AA amyloidosis was the second most common subtype, representing 18.6% (13/70) of cases. The underlying condition was Familial Mediterranean Fever (FMF) in six patients, while systemic lupus erythematosus (SLE), juvenile rheumatoid arthritis, and intravenous (IV) drug abuse were each identified as the primary disease in one patient. No underlying cause could be identified in the remaining four patients with AA amyloidosis. ATTR amyloidosis was identified in 4.3% (3/70) of patients. The amyloidosis subtype remained unclassified in 21.4% of the study population.

The median time from the onset of the first symptoms of systemic amyloidosis to diagnosis was 6.5 months (range, 1–48 months). Disease modifying treatment for specific amyloidosis subtype was administered to 64.3% of the study population and was discontinued in 2.9% of patients because of treatment intolerance, details are summarized in Table 5. Management of cardiac complications and comorbidities primarily consisted of diuretic therapy, with 70% of patients receiving loop diuretics with or without mineralocorticoid receptor antagonists (MRAs). Other commonly prescribed medications included anticoagulants, sodium–glucose cotransporter 2 inhibitors (SGLT2i), and midodrine, which were used in 20.0%, 12.9%, and 8.6% of patients, respectively.

**Table 5:** Treatment of study population

Treatment received	No (%)
**Disease modifying treatment for specific amyloidosis subtype** None Bortezomib Cyclophosphamide Dexamethasone Lenalidomide ASCT Treatment of primary disease (AA type) Tafamidis Stopped treatment due to intolerability Died before starting therapy Refused to receive specific therapy	25 (35.7) 33 (47.1) 32 (45.7) 32 (45.7) 1 (1.4) 3 (4.3) 10 (14.3) 0 (0.0) 2 (2.9) 2 (2.9) 2 (2.9)

AA: serum amyloid A amyloidosis; ASCT: autologous stem cell transplantation

### Follow up data

New complications developed in 21.4% of patients, with renal complications being the most common. Worsening renal function occurred in 5.7% of cases, while progression to end-stage renal disease (ESRD) requiring maintenance hemodialysis was observed in 7.1%. Other complications included severe anemia requiring blood transfusion (2.9%), venous thromboembolism (4.3%), systemic embolization (2.9%), worsening hepatic function (2.9%), pulmonary amyloidosis (1.4%), new onset AF (1.4%) and seizures (1.4%).

The mortality rate in our study was high, with 48.6% of patients dying during the study period. The mean time to death was 4.79 ± 4.49 months, ranging from 1 week to 17 months after diagnosis. Cardiac causes, including sudden cardiac death and progressive heart failure, were the leading cause of mortality, accounting for 38.3% of deaths. This was followed by progression of systemic amyloidosis with multiorgan failure (26.5%) and sepsis (20.6%). The cause of death remained undetermined in 14.7% of cases.

## Discussion

This prospective observational study provides a comprehensive description of the demographic and clinical characteristics and outcomes of patients with cardiac amyloidosis at a tertiary care center in Egypt. Although cardiac amyloidosis was once regarded as an uncommon cause of heart failure, recent advances in diagnostic imaging and growing clinical awareness have revealed that the disease is substantially underrecognized and more prevalent than previously appreciated [4, 8, 9, 13]. The most prevalent subtype of cardiac amyloidosis in our cohort was AL amyloidosis, accounting for 55.7% of cases. This finding is consistent with the epidemiological pattern reported worldwide until recent years [4, 8]. However, with the introduction of non-biopsy diagnostic pathways for ATTR cardiac amyloidosis and the increasing proportion of elderly individuals in developed countries, the reported prevalence of ATTR cardiac amyloidosis has risen substantially, equaling or even surpassing that of AL amyloidosis in some regions [17]. In contrast, ATTR cardiac amyloidosis accounted for only 4.3% of cases in our cohort. This discrepancy may be explained by the limited availability of technetium-99 m PYP scintigraphy and genetic testing in Egypt, which may contribute to underdiagnosis. Additionally, the lower life expectancy of the Egyptian population compared with that of Western countries may reduce the prevalence of age-related ATTR cardiac amyloidosis [11]. 

The second most common amyloidosis subtype in our cohort was AA amyloidosis, accounting for 18.6% of cases. This finding differs from reports from developed countries, where clinically significant cardiac involvement in systemic AA amyloidosis is generally considered uncommon [4, 8, 13]. However, a nationwide survey from Japan that included 199 patients with AA amyloidosis demonstrated that cardiac involvement was not negligible, with heart failure reported in 11.6% of patients, conduction abnormalities in 3.5%, and atrial or ventricular arrhythmias in 5.5%. Notably, cardiac manifestations represented the initial clinical presentation in nine patients [18]. Because AA amyloidosis results from persistent chronic inflammation associated with autoimmune or autoinflammatory disorders, as well as chronic infections [4, 8, 18, 19], its relatively high prevalence in our study population underscores the need for early recognition and optimal control of the underlying inflammatory condition. This highlights the importance of patient education regarding regular follow-up, adherence to treatment, and timely management of chronic inflammatory diseases to potentially reduce the burden of AA amyloidosis and its cardiac complications.

The specific amyloidosis subtype remained unclassified in 21.4% of the study population owing to patient death before completion of the diagnostic workup, unavailability of certain diagnostic modalities, or refusal to undergo further diagnostic evaluation. Failure to identify the specific subtype of cardiac amyloidosis is not uncommon, even in large registries, reflecting the evolving understanding of the disease [4, 8, 13, 17]. However, in our study, the proportion of unclassified cases was slightly higher, likely due to limited access to certain diagnostic modalities, particularly those required for rare CA subtypes, as well as the inability to fully implement the recommended diagnostic algorithms. A relevant example from our study is a patient who exhibited grade 3 myocardial uptake on PYP scintigraphy, an abnormal serum FLC ratio and detectable monoclonal protein on laboratory testing. Current diagnostic algorithms recommend endomyocardial biopsy for amyloid subtyping in such cases [8]. However, this could not be performed because of the unavailability of cardiac biopsy kits and the lack of transthyretin-specific antibodies required for histopathological confirmation.

During the study period, several significant diagnostic challenges were identified and can be summarized as follows:1) PYP scintigraphy is available in only a limited number of centers across Egypt, with inconsistent availability of radiotracers; 2) unavailability of cardiac biopsy performing kits; 3) immunohistochemistry remains the only available modality for histological typing and identification of specific amyloidosis subtypes, and is accessible in only a limited number of pathology laboratories; 4) genetic testing required for the diagnosis of hereditary amyloidosis variants is limited in availability and associated with substantial cost and 5) limited awareness among healthcare professionals regarding the clinical red flags of cardiac amyloidosis and currently available therapeutic options. In 2023, the authors of a paper on the diagnosis of cardiac amyloidosis in Egypt proposed an alternative diagnostic algorithm designed for resource-constrained settings, considering both the limited availability of specific diagnostic modalities and the costs associated with establishing the diagnosis [11]. Despite being authored by Egyptian experts, the paper has several important limitations. It relied primarily on epidemiological data derived from Western populations, assumed the availability of biopsy subtyping techniques for accurate amyloidosis typing, and considered extracardiac biopsy sites to have higher diagnostic sensitivity. However, real-world experience has demonstrated that this proposed diagnostic algorithm is not readily applicable in our setting because of the aforementioned challenges. When conducting studies to determine the prevalence of a newly recognized and presumed rare disease within a country, it is essential that the full range of fundamental diagnostic modalities be available. Without these resources, the entire disease spectrum may not be accurately identified, potentially leading to an underestimation of the true disease burden.

The mean age of the study population was 54.37 ± 13.85 years (range, 19–85 years), with 77% of patients being younger than 65 years. This cohort was slightly younger than those reported in most studies from developed countries, where patients with AL amyloidosis have a median age of approximately 60 years at diagnosis, while ATTR amyloidosis typically shows a peak incidence between the seventh and ninth decades of life, depending on the subtype (hereditary or wild-type) and the specific genetic mutation [4, 20–23]. This difference may be attributable to the unique epidemiological characteristics of our study population, which exhibited a lower prevalence of TTR amyloidosis and a higher prevalence of AL and AA amyloidosis than those reported in cohorts from developed countries.

Disease modifying treatment for specific amyloidosis subtype was given to 64.3% of the study population in the form of standard triple regimen with or without autologous stem cell transplantation (ASCT) in AL type and anti-inflammatory, immunosuppressants and biological therapy in AA type [4, 8, 13]. None of the three patients diagnosed with TTR amyloidosis in our cohort received tafamidis. The reasons included advanced disease stage in one patient, financial constraints in another, and patient refusal of therapy in the third. Although tafamidis has been shown to improve clinical outcomes in patients with ATTR cardiac amyloidosis, its use remains limited in real-world practice. Barriers include the high cost of treatment, the advanced age at diagnosis of many patients, and regulatory policies in many countries that restrict its use to patients with NYHA functional class I–II, resulting in many eligible patients remaining untreated [22, 24–26].

Nearly half of the study population (48.6%) died during the follow-up period. This high mortality rate likely reflects the advanced stage of cardiac disease at the time of diagnosis. Specifically, 61.5% of patients presented with NYHA functional class III-IV symptoms despite receiving outpatient diuretic therapy, and 47.1% were hypotensive at presentation. Furthermore, low QRS voltage disproportionate to the degree of left ventricular wall thickness was observed in 75.7% of patients, while the mean NT-proBNP level was markedly elevated (14,146.5 ± 11,228.6 pg/mL). These features are recognized indicators of advanced cardiac involvement and have been consistently identified as predictors of adverse clinical outcomes and mortality [8, 13, 22, 23].

### Study strengths and limitations

To the best of our knowledge, this is the first study to report the epidemiological characteristics of cardiac amyloidosis in an Egyptian cohort. However, several limitations should be acknowledged. First, the relatively small sample size and the heterogeneous study population, comprising different amyloidosis subtypes, limited our ability to identify independent predictors of mortality. Second, this was a single-center study, which may limit the generalizability of our findings. Nevertheless, our tertiary referral center serves four major governorates, making the study population reasonably representative of patients with cardiac amyloidosis in Egypt. Larger, multicenter studies are warranted to validate our findings. Third, the limited availability of certain advanced diagnostic modalities in Egypt prevented definitive amyloid subtype identification in a subset of patients, which may have affected the precision of subtype-specific analyses.

## Conclusions

This tertiary care center study of cardiac amyloidosis in Egyptian patients demonstrated a substantial diagnostic delay and poor clinical outcomes. In our study cohort, AL amyloidosis was the most common subtype, while the specific subtype couldn’t be identified in about one fifth of the study population due to limited diagnostic resources or early mortality. Nearly half of patients died during the study period, highlighting the advanced stage of disease at presentation and its grave prognosis. These findings underscore the need for greater clinical awareness, streamlined diagnostic pathways and improved access to comprehensive diagnostic modalities to ensure timely diagnosis and optimize patient outcomes. Larger multi-center studies are needed to better define the epidemiology and disease burden of cardiac amyloidosis in Egypt.

## Data Availability

The datasets used and/or analyzed during the current study are available from the corresponding author on reasonable request.

## Abbreviations

AA: Serum amyloid A amyloidosis
ACEIs: Angiotensin-converting enzyme inhibitors
AF: Atrial fibrillation
AL: Light chain amyloidosis
ARBs: Angiotensin receptor blockers
ARNIs: Angiotensin receptor-neprilysin inhibitors
ATTR: Transthyretin amyloidosis
AV: Atrioventricular
BMI: Body mass index
BP: Blood pressure
CA: Cardiac amyloidosis
CE-CMR: Contrast enhanced cardiac magnetic resonance
CHB: Complete heart block
CI: Confidence interval
COPD: Chronic obstructive pulmonary disease
CTS: Carpel tunnel syndrome
CVS: Cerebrovascular stroke
DVT: Deep venous thrombosis
DOACs: Direct oral anticoagulants
ECG: Electrocardiogram
ECV: Extracellular volume
EF: Ejection fraction
eGFR: estimated glomerular filtration rate
ESRD: End stage renal disease
FLC: Free light chain
FMF: Familial mediterranean fever
GLS: Global longitudinal strain
Hb: Hemoglobin
HF: Heart failure
HR: Heart rate
IAS: Interatrial septum
ILF: Interstitial lung fibrosis
IV: Intravenous
IVCD: Intraventricular conduction delay
IVSd: Interventricular septal diameter
LA: Left atrium
LAVi: Left atrial volume index
LBBB: Left bundle branch block
LGE: Late gadolinium enhancement
LL: Lower limb
LV: Left ventricle
LVH: Left ventricular hypertrophy
MRA: Mineralocorticoid receptor antagonist
NYHA: New york heart association
OR: Odds ratio
PACs: Premature atrial contractions
PE: Pulmonary embolism
proBNP: pro-B-type brain natriuretic peptides
PVCs: Premature ventricular contractions
PWT: Posterior wall thickness
PYP: Pyrophosphate
QTc: Heart-rate corrected QT interval
RA: Rheumatoid arthritis
RV: Right ventricle
RVH: Right ventricular hypertrophy
SD: Standard deviation
SGLT2i: Sodium-glucose cotransporter 2 inhibitor
SLE: Systemic lupus erythematosus
STE: Speckle tracking echocardiography
TIA: Transient ischemic attack
ULN: Upper limit normal
WBCs: White blood cells
VKA: Vitamin K antagonist
